# Supplementary material for: Integrated multi-omics reveals cellular and molecular interactions governing the invasive niche of basal cell carcinoma
Source: Nat Commun. 2022 Aug 20;13:4897. doi: 10.1038/s41467-022-32670-w (PMC9391376; doi:10.1038/s41467-022-32670-w)
Supplement: Supplementary file 1 — Supplementary Information [file 41467_2022_32670_MOESM1_ESM.pdf]

Integrated multi-omics reveals cellular and molecular interactions governing the invasive niche of basal cell carcinoma

SUPPLEMENTARY INFORMATION

a

ID	Diagnosis	Location	Previous treatment	Sample
BCC1	infiltrative BCC	left ear	no	scRNA-seq
BCC2	infiltrative BCC	forehead	no	scRNA-seq
BCC3	infiltrative BCC	left cheek	no	scRNA-seq
BCC4	infiltrative BCC	nose	no	scRNA-seq
BCC5	infiltrative BCC	nose	no	scRNA-seq
DSP1	nodular BCC	right leg	no	DSP-seq
DSP2	nodular BCC	scalp	no	DSP-seq
DSP3	nodular BCC	left leg	no	DSP-seq
DSP4	nodular BCC	upper lip	no	DSP-seq
DSP5	nodular BCC	forehead	no	DSP-seq
DSP6	nodular BCC	right cheek	no	DSP-seq
DSP7	infiltrative BCC	left eyebrow	no	DSP-seq
DSP8	infiltrative BCC	right elbow	no	DSP-seq
DSP9	infiltrative BCC	left ear	no	DSP-seq
DSP10	infiltrative BCC	left ear	no	DSP-seq
DSP11	infiltrative BCC	left cheek	no	DSP-seq
DSP12	infiltrative BCC	nose	no	DSP-seq

b

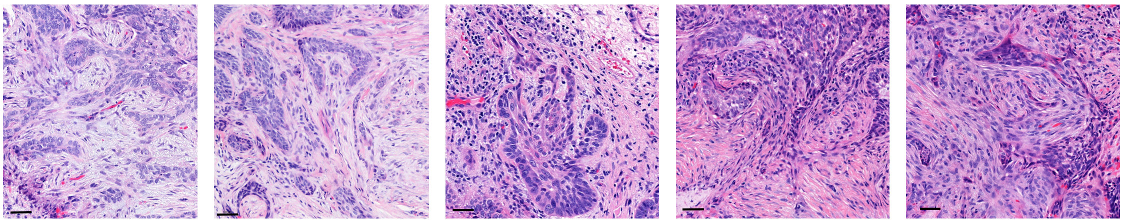

c

ID	# cells before filtering	# cells after filtering	# filtered out cells	% filtered out cells
BCC1	5856	5475	381	6.51
BCC2	9109	8562	547	6.01
BCC3	3823	3553	270	7.06
BCC4	6728	6181	547	8.13
BCC5	5267	5039	228	4.33

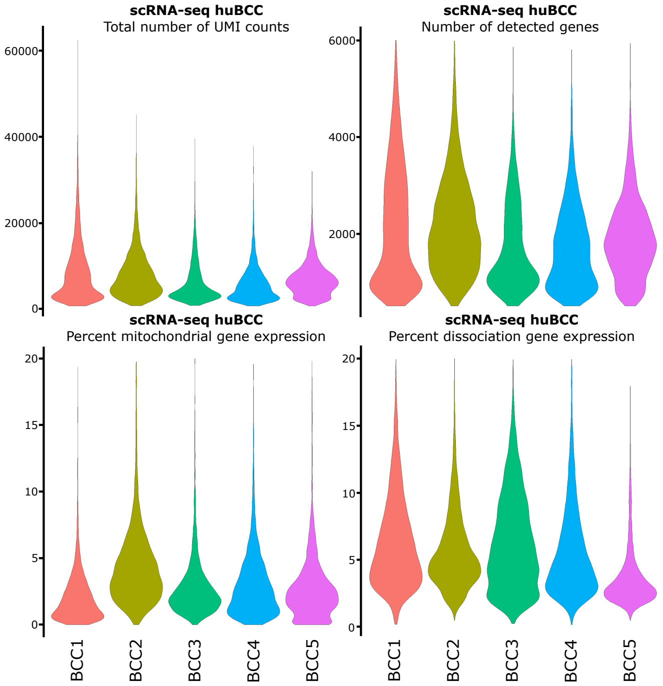

d

Cell subtype	Number of cells	Percentage
Epithelial cells	14586	50.63
T cells	6565	22.79
Melanocytes	2440	8.47
Myeloid cells	1384	4.80
Endothelial cells	943	3.27
Fibroblasts	809	2.81
Cycling cells	560	1.94
Pericytes	486	1.69
Mast cells	439	1.52
B cells	598	2.08
Total	28810	100.00

e

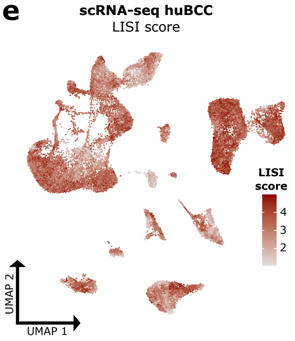

f

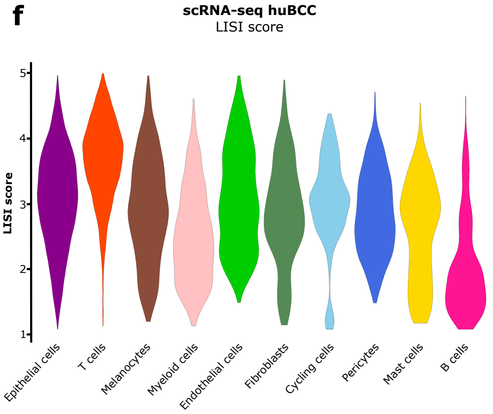

Supplementary Fig. 1 Characteristics of tumor samples and quality controls of scRNA-seq.

a) Table representing the characteristics of the five samples used for scRNA-seq and the twelve samples used for DSP analysis. Patient ages ranged from 49 to 92 years old. **b)** Histology of the five infiltrative human BCCs (huBCCs) selected for scRNA-seq analysis. Scale bars indicate 50 µm. **c)** Table representing the number of cells from each huBCC scRNA-seq sample before and after filtering for low quality cells, the number of filtered out cells and the proportion of filtered out cells relative to the initial number of cells. Violin plots show the total number of UMI counts per cell (upper left), number of detected genes per cell (upper right), percentage of mitochondrial gene expression per cell (lower left) and the percentage of dissociation-associated genes expression per cell (lower right) after integration of the five filtered scRNA-seq huBCC samples. **d)** Table indicating the total number of cells present in individual cell type subpopulations and the proportion of individual subpopulations relative to the total amount of cells in the scRNA-seq dataset. **e)** UMAP plot showing the Local Inverse Simpson's Index (LISI) score of five filtered scRNA-seq huBCC samples after integration. **f)** Violin plots illustrating the LISI score of each cell type subpopulations after integration of the five filtered scRNA-seq huBCC samples.

BCC, basal cell carcinoma; DSP, digital spatial profiling; scRNA-seq, single-cell RNA sequencing; DSP-seq, digital spatial profiling sequencing; #, number; %, percent; huBCC, human basal cell carcinoma; UMI, unique molecular identifier; LISI, local inverse Simpson's index.

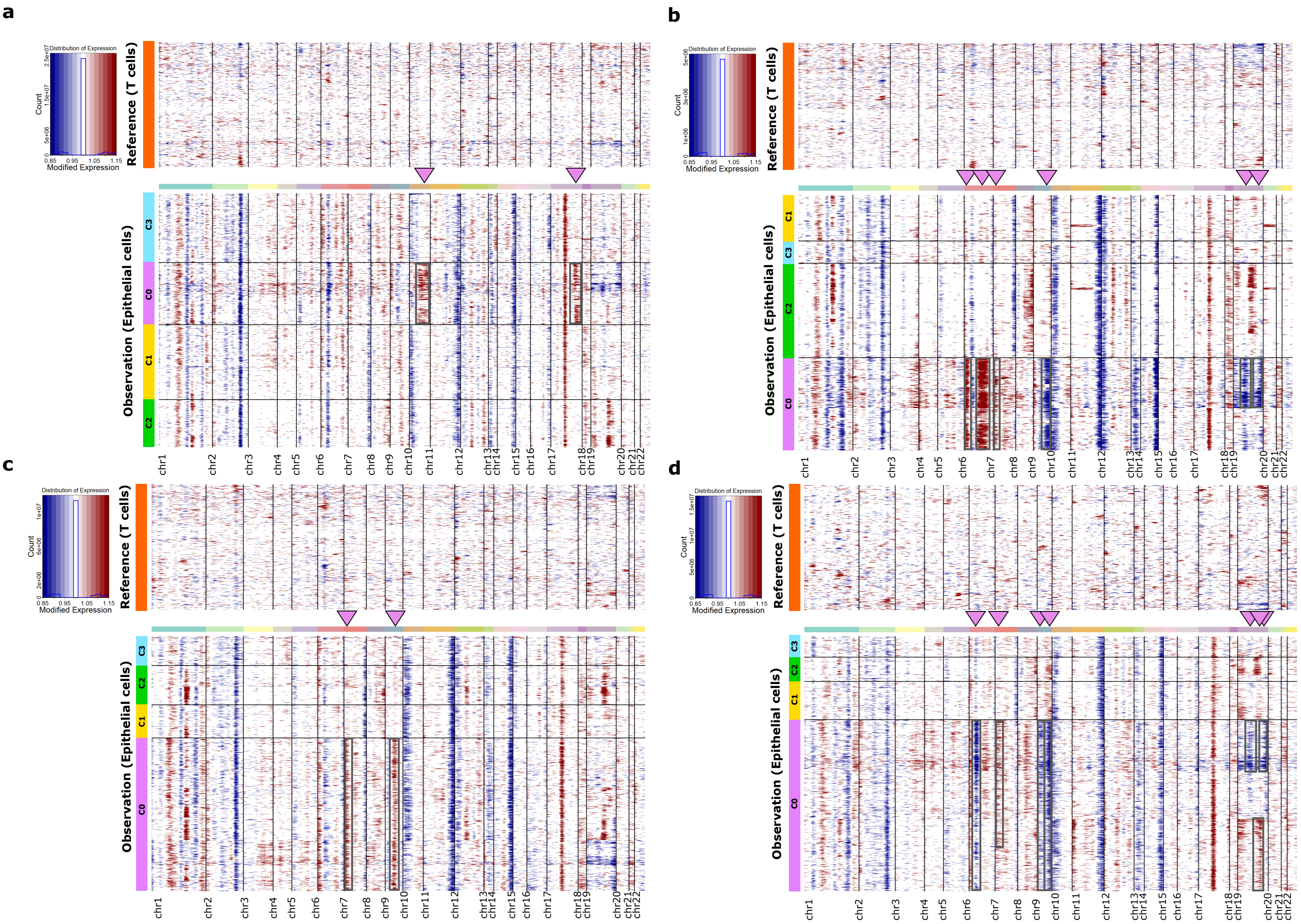
a
b
c
d
Distribution of Expression
Count
Modified Expression
0.85
0.95
1.05
1.15
Reference (T cells)
Reference
Observation (Epithelial cells)
C0
C1
C2
C3
chr1
chr2
chr3
chr4
chr5
chr6
chr7
chr8
chr9
chr10
chr11
chr12
chr13
chr14
chr15
chr16
chr17
chr18
chr19
chr20
chr21
chr22

Supplementary Fig. 2 Single cell copy-number variation analysis of *KRT14*^{pos} epithelial cells.

Representative CNV heatmaps from inferCNV analysis of *KRT14*^{pos} epithelial cell clusters compared to T cells as reference for BCC2 **(a)**, BCC3 **(b)**, BCC4 **(c)**, and BCC5 **(d)**. Arrowheads and rectangles highlight inferred CNVs, after exclusion of gene expression changes shared by the *KRT14*^{pos} epithelial cells from the five BCC samples.

BCC, basal cell carcinoma; CNV, copy number variation.

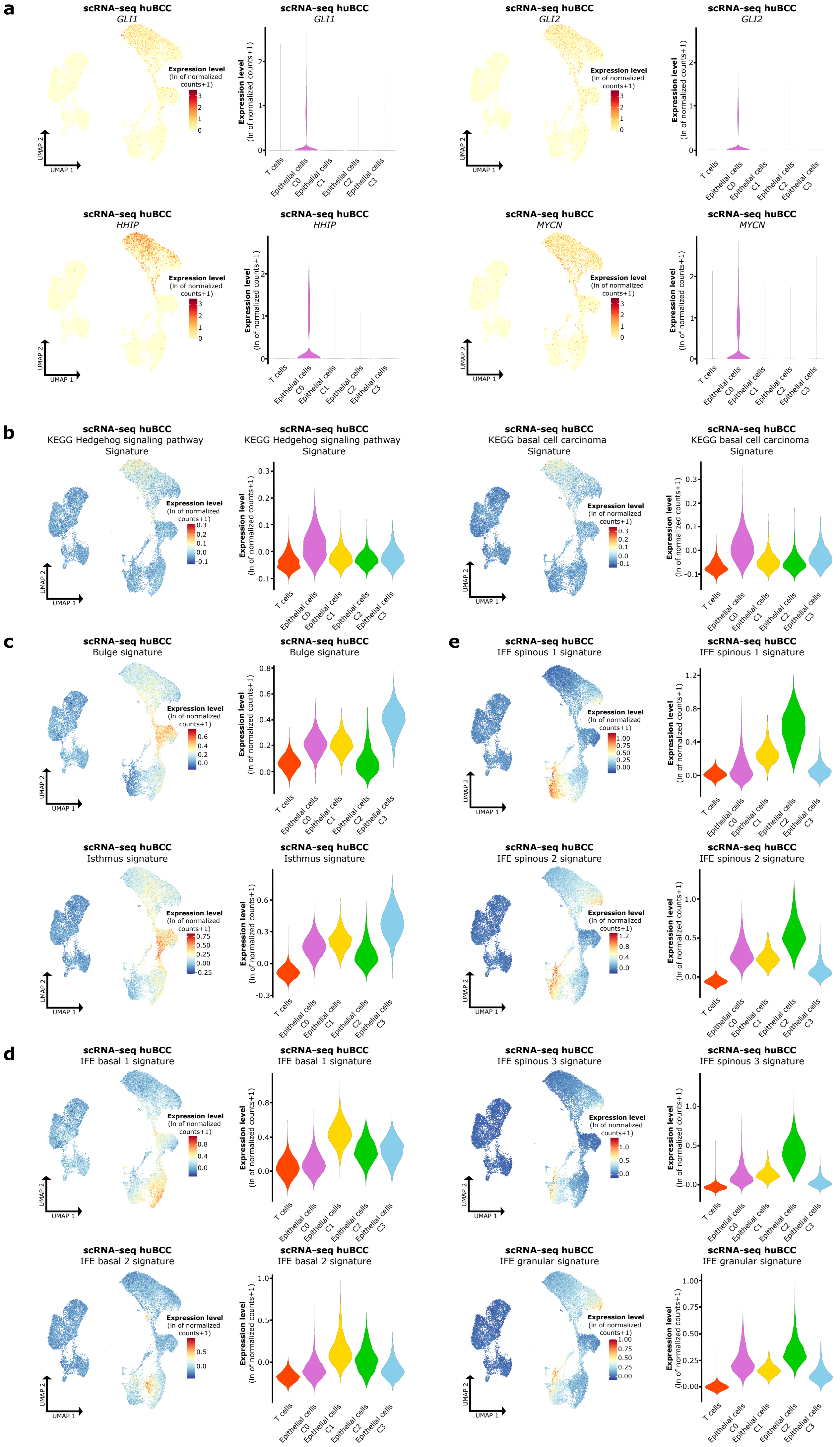
a
scRNA-seq huBCC
GLI1
GLI2
HHIP
MYCN
Expression level
(ln of normalized counts+1)
UMAP 1
UMAP 2
T cells
Epithelial cells C0
Epithelial cells C1
Epithelial cells C2
Epithelial cells C3
b
KEGG Hedgehog signaling pathway Signature
KEGG basal cell carcinoma Signature
c
Bulge signature
Isthmus signature
d
IFE basal 1 signature
IFE basal 2 signature
e
IFE spinous 1 signature
IFE spinous 2 signature
IFE spinous 3 signature
IFE granular signature

Supplementary Fig. 3 Characterization of the non-cycling *KRT14*^{pos} epithelial cell subpopulations in scRNA-seq.

a) Expression level of BCC (*GLI1, GLI2, HHIP, MYCN*) marker genes in the epithelial and T cell subpopulations, represented as a color scale overlaid on the UMAP plot (left panels) and as violin plots per cell type (right panels). **b)** Average expression level of the “KEGG Hedgehog signaling pathway” (left panels) and “KEGG basal cell carcinoma” (right panels) signatures in the epithelial and T cell subpopulations, represented as a color scale overlaid on the UMAP plot and as violin plots per cell type. **c)** Average expression level of the “hair follicle” signatures in the epithelial and T cell subpopulations, represented as a color scale overlaid on the UMAP plot (left panels) and as violin plots per cell type (right panels). **d)** Average expression level of the “basal IFE” signatures in the epithelial and T cell subpopulations, represented as a color scale overlaid on the UMAP plot (left panels) and as violin plots per cell type (right panels). **e)** Average expression level of “spinous IFE” and “granular IFE” signatures in the epithelial and T cell subpopulations, represented as a color scale overlaid on the UMAP plot (left panels) and as violin plots per cell type (right panels).

scRNA-seq, single-cell RNA sequencing; huBCC, human basal cell carcinoma; IFE, interfollicular epidermis.

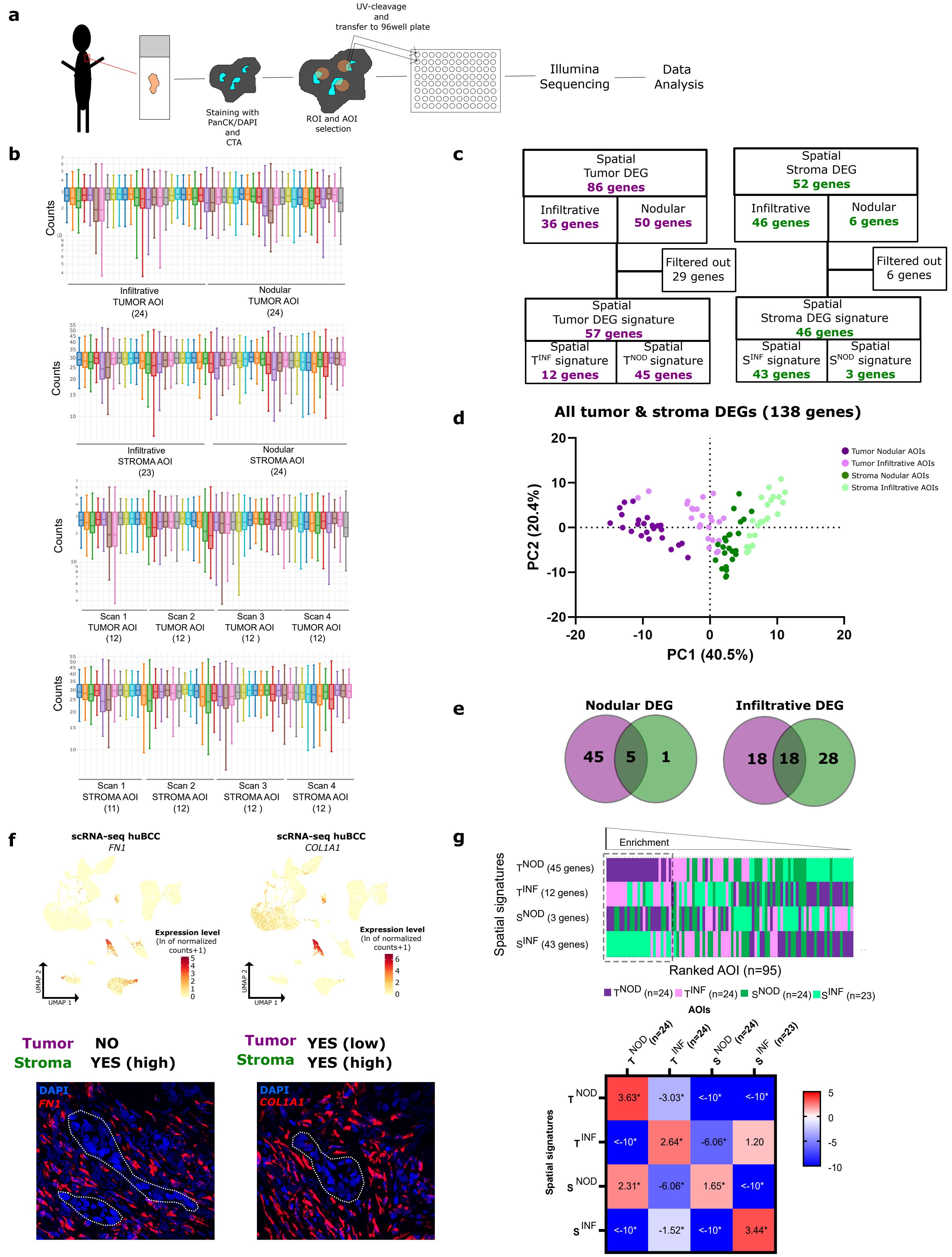

a
UV-cleavage and transfer to 96well plate
Staining with PanCK/DAPI and CTA
ROI and AOI selection
Illumina Sequencing
Data Analysis
b
Counts
Infiltrative TUMOR AOI (24)
Nodular TUMOR AOI (24)
Infiltrative STROMA AOI (23)
Nodular STROMA AOI (24)
Scan 1 TUMOR AOI (12)
Scan 2 TUMOR AOI (12)
Scan 3 TUMOR AOI (12)
Scan 4 TUMOR AOI (12)
Scan 1 STROMA AOI (11)
Scan 2 STROMA AOI (12)
Scan 3 STROMA AOI (12)
Scan 4 STROMA AOI (12)
c
Spatial Tumor DEG 86 genes
Infiltrative 36 genes
Nodular 50 genes
Filtered out 29 genes
Spatial Tumor DEG signature 57 genes
Spatial T^INF signature 12 genes
Spatial T^NOD signature 45 genes
Spatial Stroma DEG 52 genes
Infiltrative 46 genes
Nodular 6 genes
Filtered out 6 genes
Spatial Stroma DEG signature 46 genes
Spatial S^INF signature 43 genes
Spatial S^NOD signature 3 genes
d
All tumor & stroma DEGs (138 genes)
Tumor Nodular AOIs
Tumor Infiltrative AOIs
Stroma Nodular AOIs
Stroma Infiltrative AOIs
PC2 (20.4%)
PC1 (40.5%)
e
Nodular DEG
45 5 1
Infiltrative DEG
18 18 28
f
scRNA-seq huBCC FN1
scRNA-seq huBCC COL1A1
Expression level (ln of normalized counts+1)
UMAP 1
UMAP 2
Tumor NO
Stroma YES (high)
Tumor YES (low)
Stroma YES (high)
DAPI FN1
DAPI COL1A1
g
Enrichment
Spatial signatures
T^NOD (45 genes)
T^INF (12 genes)
S^NOD (3 genes)
S^INF (43 genes)
Ranked AOI (n=95)
T^NOD (n=24) T^INF (n=24) S^NOD (n=24) S^INF (n=23)
AOIs
T NOD (n=24)
T INF (n=24)
S NOD (n=24)
S INF (n=23)
T NOD: 3.63* -3.03* <-10* <-10*
T INF: <-10* 2.64* -6.06* 1.20
S NOD: 2.31* -6.06* 1.65* <-10*
S INF: <-10* -1.52* <-10* 3.44*

Supplementary Fig. 4 Establishment of compartment-specific DEG signatures for nodular and infiltrative tumor-stroma interfaces.

a) Schematic of the GeoMx DSP experimental process from tissue collection to data analysis. **b)** Box plots representing the normalized counts per AOI depending on tumor subtype (two upper panels) or scan processing number (two lower panels). **c)** Graphical representation of the establishment of spatial signatures. **d)** PCA plot of the enrichment of all tumor and stroma DEGs in all tumor and stroma AOIs.**e)** Venn diagrams showing the overlap between DEGs in the tumor (purple) and stroma AOIs (green) for each BCC subtype. **f)** Tumor and stromal expression of *FN1* and *COL1A1* determined on scRNA-seq and RNA FISH, illustrating tumor-stroma RNA cross-contamination. Dotted lines highlight the tumor-stroma interface. Scale bars indicate 20 µm. **g)** Ranked individual AOIs according to their enrichment for specific spatial signatures (upper panel). Dotted lines indicate upper quartile (25%) with the highest enrichment. Specificity of individual spatial signatures assessed by the representation of subtype-specific AOI in the 25% most enriched AOIs (lower panel). Positive and negative values indicate over- and under-representation respectively compared to expectations (fold change), based on the cumulative distribution function of the hypergeometric distribution. * p-value <0.05.

panCK, pan-cytokeratin; CTA, cancer transcriptome atlas; ROI, region of interest; AOI, area of interest; UV-cleavage, ultra-violet cleavage; DEGs, differentially expressed genes; T^{NOD}, nodular tumor; T^{INF}, infiltrative tumor; S^{NOD}, nodular stroma; S^{INF}; infiltrative stroma; PC, principal component; PCA, principal component analysis; scRNA-seq, single-cell RNA sequencing; huBCC, human basal cell carcinoma.

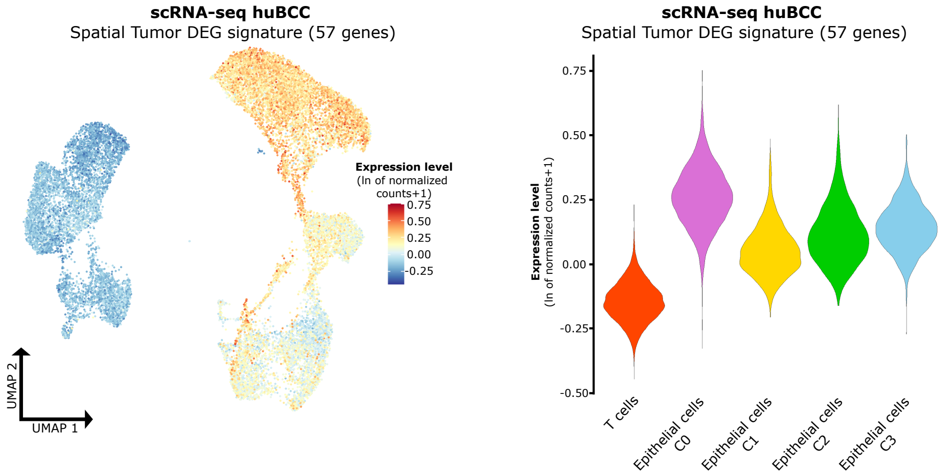
scRNA-seq huBCC
Spatial Tumor DEG signature (57 genes)
Expression level
(ln of normalized counts+1)
0.75
0.50
0.25
0.00
-0.25
UMAP 2
UMAP 1
scRNA-seq huBCC
Spatial Tumor DEG signature (57 genes)
Expression level
(ln of normalized counts+1)
0.75
0.50
0.25
0.00
-0.25
-0.50
T cells
Epithelial cells C0
Epithelial cells C1
Epithelial cells C2
Epithelial cells C3

Supplementary Fig. 5 Spatial signatures map cell subtypes composing tumor-stroma interface in infiltrative BCCs.

Average expression level of the spatial Tumor DEG signature in epithelial cells (tumor cells and keratinocytes) and T cells of five huBCC samples, represented as a color scale overlaid on the UMAP plot (left panel) and as violin plots per cell type (right panel).

scRNA-seq, single-cell RNA sequencing; huBCC, human basal cell carcinoma; DEG, differentially expressed genes; UMAP, uniform manifold approximation and projection; C, cluster.

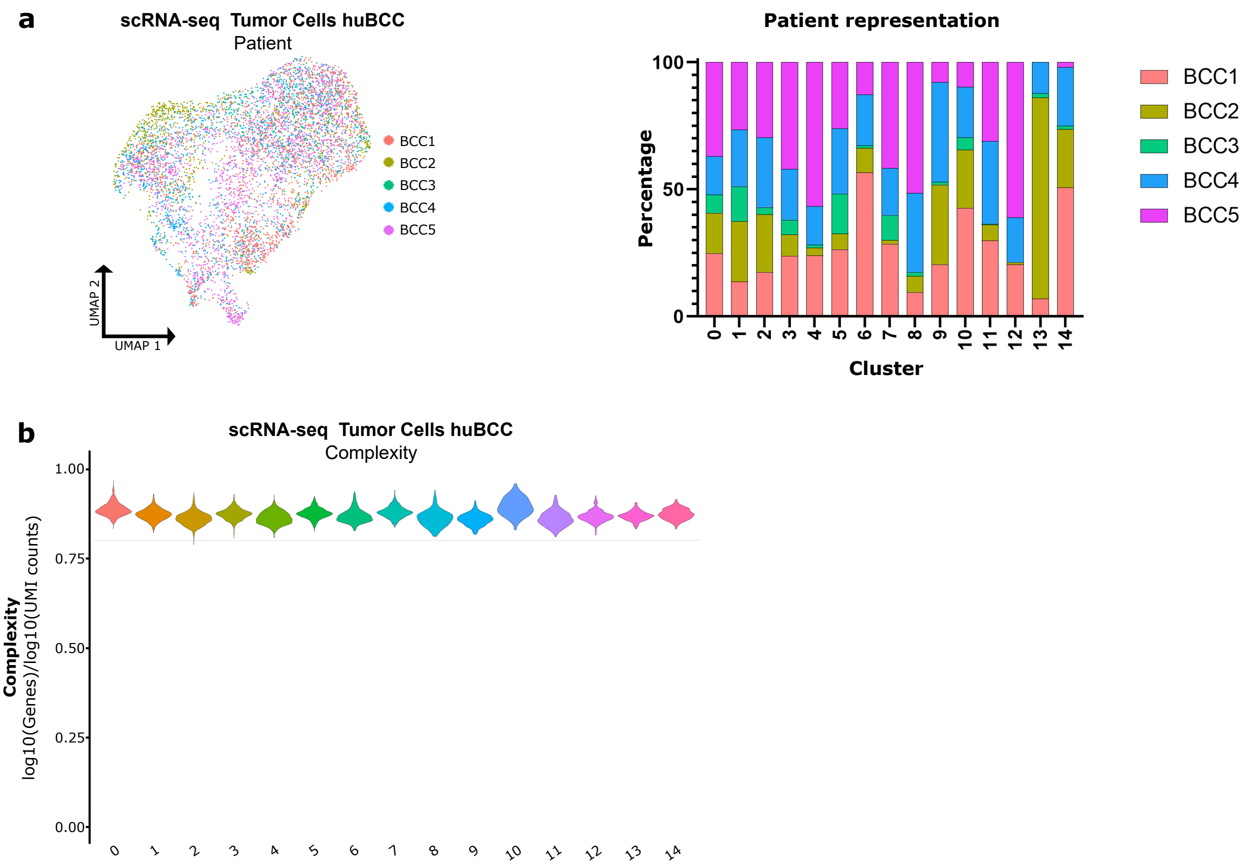
a
scRNA-seq Tumor Cells huBCC
Patient
BCC1
BCC2
BCC3
BCC4
BCC5
UMAP 2
UMAP 1
Patient representation
Percentage
100
50
0
0 1 2 3 4 5 6 7 8 9 10 11 12 13 14
Cluster
b
scRNA-seq Tumor Cells huBCC
Complexity
Complexity
log10(Genes)/log10(UMI counts)
1.00
0.75
0.50
0.25
0.00

Supplementary Fig. 6 Quality control of the scRNA-seq tumor cell subpopulations.

a) UMAP plot of the tumor cell subpopulations colored according to the huBCC samples (left panel) and bar graph of the scRNA-seq tumor cell subpopulations illustrating the contribution by sample to each cluster (right panel). **b)** Complexity of gene expression in each cluster of the scRNA-seq tumor cell subpopulations, represented as violin plots. Dotted line indicates the expected inferior threshold for satisfactory complexity (0.8).

scRNA-seq, single-cell RNA sequencing; huBCC, human basal cell carcinoma; BCC, basal cell carcinoma; UMAP, uniform manifold approximation and projection; UMI, unique molecular identifier.

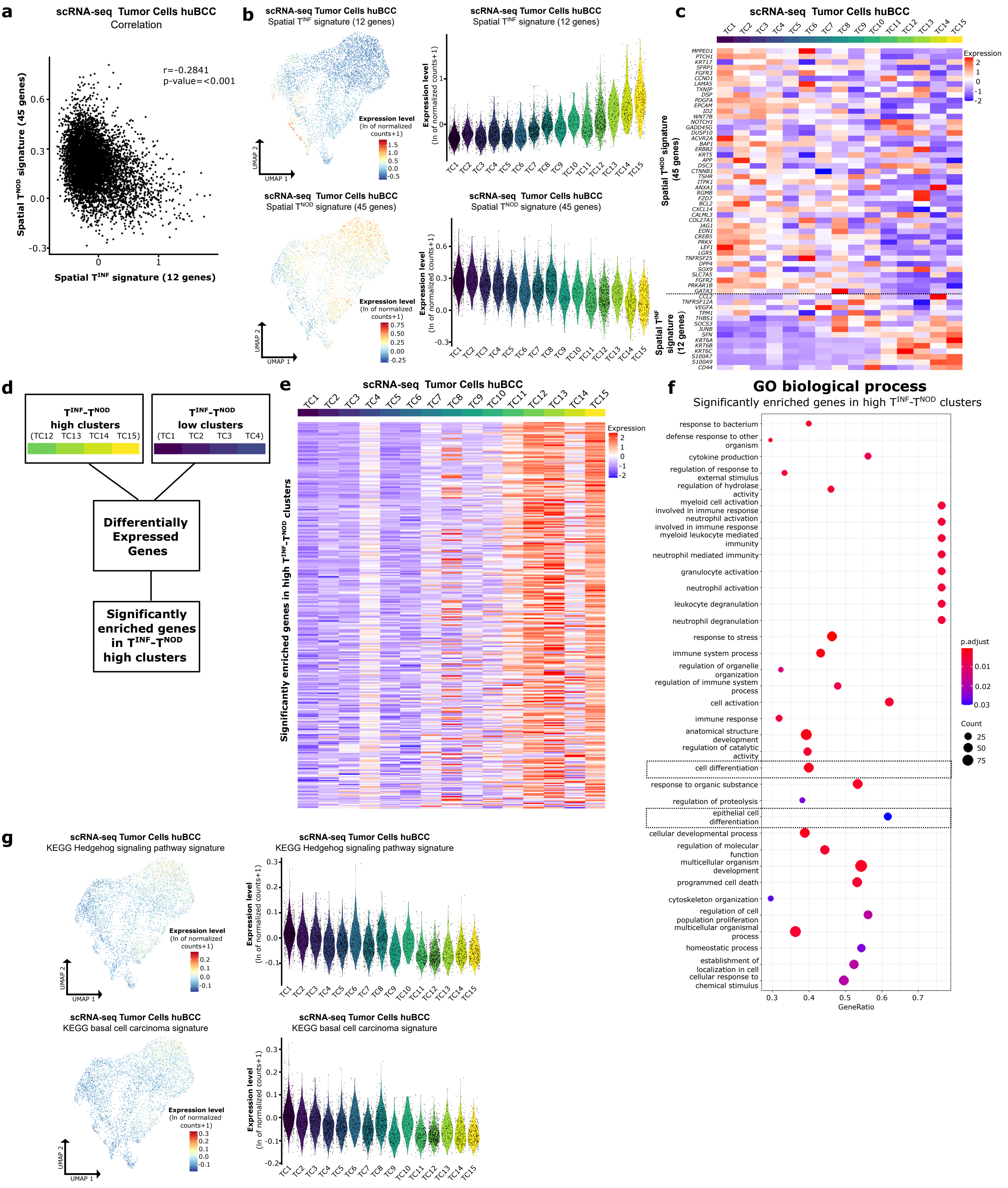
a
scRNA-seq Tumor Cells huBCC
Correlation
r=-0.2841
p-value=<0.001
Spatial T^NOD signature (45 genes)
Spatial T^INF signature (12 genes)
b
scRNA-seq Tumor Cells huBCC
Spatial T^INF signature (12 genes)
Expression level
(ln of normalized counts+1)
UMAP 1
UMAP 2
scRNA-seq Tumor Cells huBCC
Spatial T^NOD signature (45 genes)
TC1 TC2 TC3 TC4 TC5 TC6 TC7 TC8 TC9 TC10 TC11 TC12 TC13 TC14 TC15
c
scRNA-seq Tumor Cells huBCC
Expression
Spatial T^NOD signature (45 genes)
MPPED1 PTCH1 KRT17 SFRP1 FGFR3 CCND1 LAMA5 TXNIP DSP PDGFA EPCAM ID2 WNT7B NOTCH1 GADD45G DUSP10 ACVR2A BAP1 ERBB2 KRT5 APP DSC3 CTNNB1 TSHR ITPK1 ANXA1 RGMB FZD7 BCL2 CXCL14 CALML3 COL27A1 JAG1 EDN1 CREB5 PRKX LEF1 LGR5 TNFRSF25 DPP4 SOX9 SLC7A5 FGFR2 PRKAR1B GATA3
Spatial T^INF signature (12 genes)
CCL2 TNFRSF12A VEGFA TPM1 THBS1 SOCS3 JUNB SFN KRT6A KRT6B KRT6C S100A7 S100A9 CD44
d
T^INF-T^NOD high clusters
(TC12 TC13 TC14 TC15)
T^INF-T^NOD low clusters
(TC1 TC2 TC3 TC4)
Differentially Expressed Genes
Significantly enriched genes in T^INF-T^NOD high clusters
e
scRNA-seq Tumor Cells huBCC
Expression
Significantly enriched genes in high T^INF-T^NOD clusters
f
GO biological process
Significantly enriched genes in high T^INF-T^NOD clusters
response to bacterium
defense response to other organism
cytokine production
regulation of response to external stimulus
regulation of hydrolase activity
myeloid cell activation involved in immune response
neutrophil activation involved in immune response
myeloid leukocyte mediated immunity
neutrophil mediated immunity
granulocyte activation
neutrophil activation
leukocyte degranulation
neutrophil degranulation
response to stress
immune system process
regulation of organelle organization
regulation of immune system process
cell activation
immune response
anatomical structure development
regulation of catalytic activity
cell differentiation
response to organic substance
regulation of proteolysis
epithelial cell differentiation
cellular developmental process
regulation of molecular function
multicellular organism development
programmed cell death
cytoskeleton organization
regulation of cell population proliferation
multicellular organismal process
homeostatic process
establishment of localization in cell
cellular response to chemical stimulus
GeneRatio
p.adjust
Count
g
scRNA-seq Tumor Cells huBCC
KEGG Hedgehog signaling pathway signature
scRNA-seq Tumor Cells huBCC
KEGG basal cell carcinoma signature
Expression level
(ln of normalized counts+1)

Supplementary Fig. 7 Characterization of tumor cell subpopulations within the invasive niche.

a) Correlation analysis between the spatial T^{INF} and spatial T^{NOD} signature enrichment scores in individual tumor cells from the scRNA-seq tumor cell subpopulations. R coefficient and p-value were calculated by Spearman correlation test and a two-sided statistical significance test respectively. **b)** Average expression level of the spatial T^{INF} signature (upper panels) and the spatial T^{NOD} signature (lower panels) in tumor cell subpopulations of the scRNA-seq dataset, represented as a color scale overlaid on the UMAP plot (left panels) and as violin plots per ranked cluster (right panels). **c)** Heatmap of the average expression level of genes present in the T^{NOD} and T^{INF} signatures in tumor cell subpopulations. **d)** Graphical representation of the establishment of the significantly enriched genes in clusters with high T^{INF}-T^{NOD} enrichment score in the tumor cell subpopulations of the scRNA-seq dataset. **e)** Heatmap of the average expression level of significantly enriched genes in tumor cell subpopulations. **f)** Dot plot showing the GO biological processes enriched in the significantly enriched genes. **g)** Average expression level of the “KEGG Hedgehog signaling pathway” and “KEGG basal cell carcinoma” signatures in tumor cell subpopulations of the scRNA-seq dataset, represented as a color scale overlaid on the UMAP plot and as violin plots per ranked cluster.

scRNA-seq, single-cell RNA sequencing; huBCC, human basal cell carcinoma; BCC, basal cell carcinoma; T^{NOD}, nodular tumor; T^{INF}, infiltrative tumor; UMAP, uniform manifold approximation and projection; TC, tumor cluster; GO, gene ontology; p.adjust, adjusted p-value.

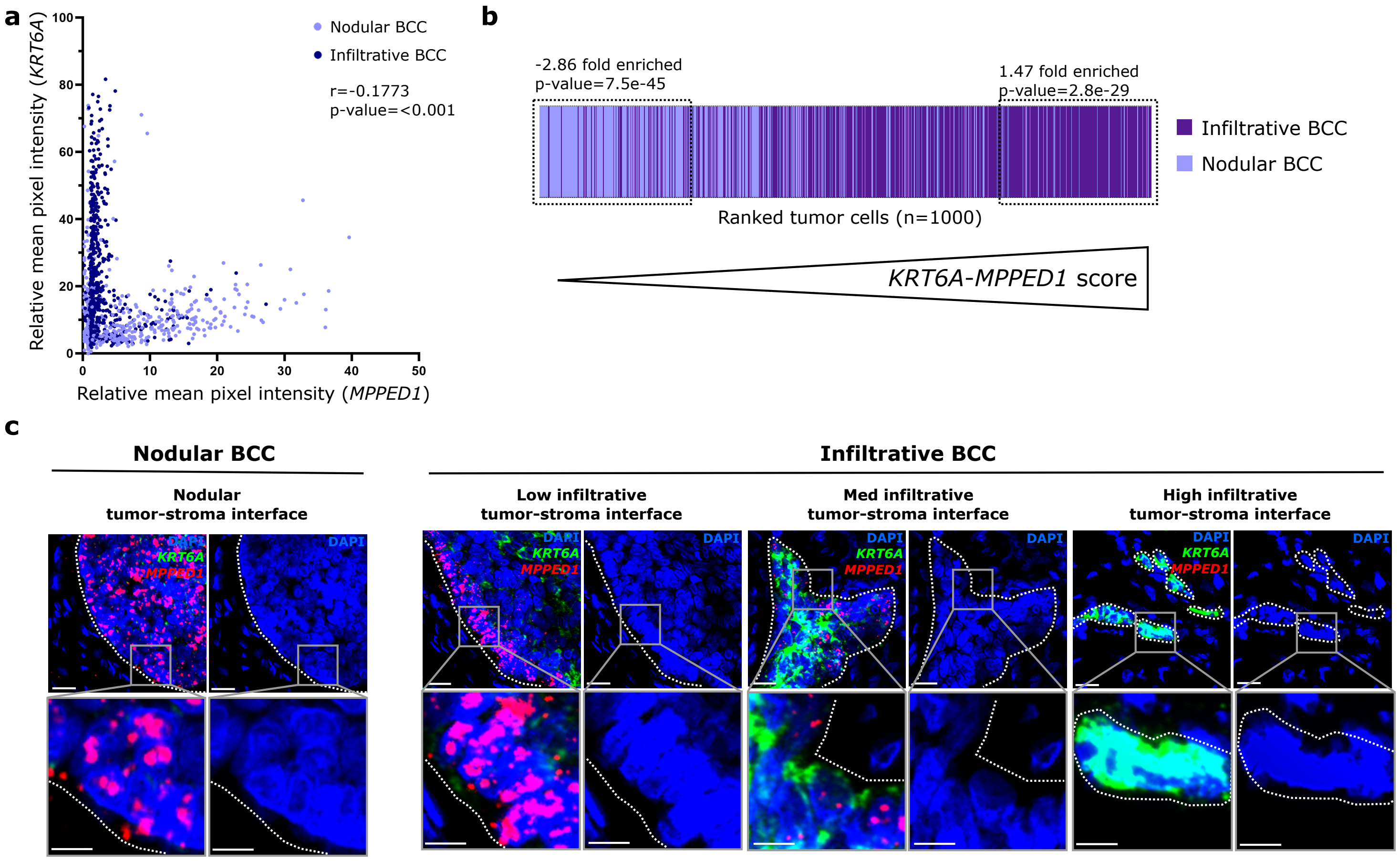
a
Nodular BCC
Infiltrative BCC
r=-0.1773
p-value=<0.001
Relative mean pixel intensity (KRT6A)
Relative mean pixel intensity (MPPED1)
0
10
20
30
40
50
60
80
100
b
-2.86 fold enriched
p-value=7.5e-45
1.47 fold enriched
p-value=2.8e-29
Infiltrative BCC
Nodular BCC
Ranked tumor cells (n=1000)
KRT6A-MPPED1 score
c
Nodular BCC
Infiltrative BCC
Nodular
tumor-stroma interface
Low infiltrative
tumor-stroma interface
Med infiltrative
tumor-stroma interface
High infiltrative
tumor-stroma interface
DAPI
KRT6A
MPPED1

Supplementary Fig. 8 *KRT6A-MPPED1* difference score in tumor cells parallels morphological changes at the tumor-stroma interface.

a) Correlation analysis between *MPPED1* and *KRT6A* relative intensities in individual tumor cells located at the tumor-stroma interface in nodular (N=4) and infiltrative (N=6) BCCs (n=100 cells/sample). R coefficient and p-value were calculated by Spearman correlation test and a two-sided statistical significance test respectively. **b)** Ranked individual cells according to the difference between *KRT6A* and *MPPED1* relative intensities. Dotted lines indicate the lower quartile (25%) (left) with the lowest difference score and the upper quartile (25%) (right) with the highest difference score. Negative and positive values indicate under- and over-representation respectively of infiltrative BCCs compared to expectation (fold change), based on the cumulative distribution function of the hypergeometric distribution. **c)** Representative scan images of Nodular, Low, Med and High infiltrative tumor-stroma interfaces stained with DAPI (blue), *KRT6A* (green), and *MPPED1* (red) probes for RNA FISH. Dotted lines highlight the tumor-stroma interface. Scale bars indicate 20 μm in upper images and 10 μm in zoom areas.

Source data for a) and b) are provided as a Source Data file.

BCC, basal cell carcinoma.

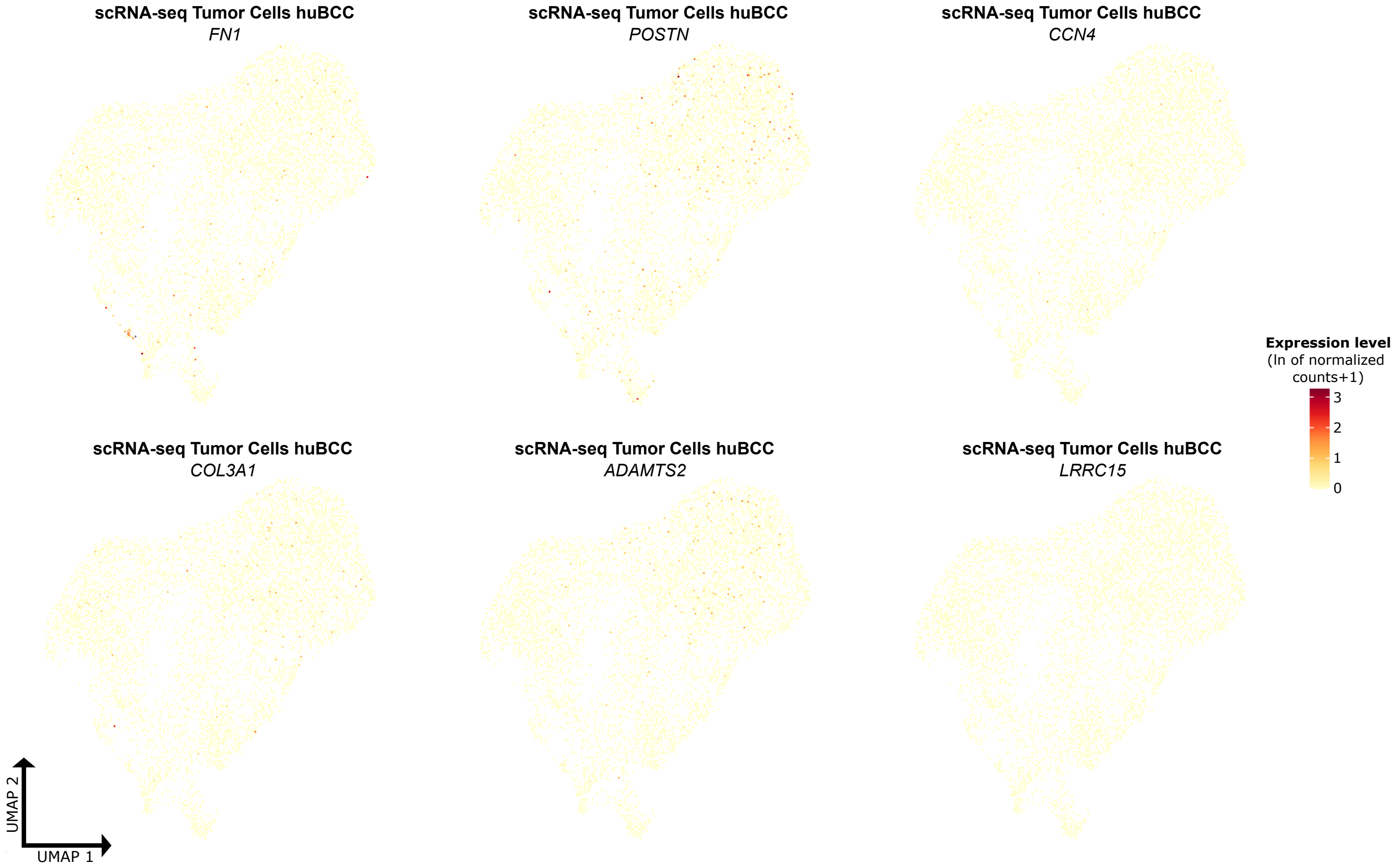
scRNA-seq Tumor Cells huBCC
FN1
scRNA-seq Tumor Cells huBCC
POSTN
scRNA-seq Tumor Cells huBCC
CCN4
scRNA-seq Tumor Cells huBCC
COL3A1
scRNA-seq Tumor Cells huBCC
ADAMTS2
scRNA-seq Tumor Cells huBCC
LRRC15
Expression level
(ln of normalized counts+1)
3
2
1
0
UMAP 2
UMAP 1

Supplementary Fig. 9 Tumor cells show little or no expression of known infiltrative BCC-specific marker genes.

Expression level of known infiltrative BCC-specific marker genes in tumor cell subpopulations of the scRNA-seq dataset, represented as a color scale overlaid on the UMAP plot.

scRNA-seq, single-cell RNA sequencing; huBCC, human basal cell carcinoma; UMAP, uniform manifold approximation and projection.

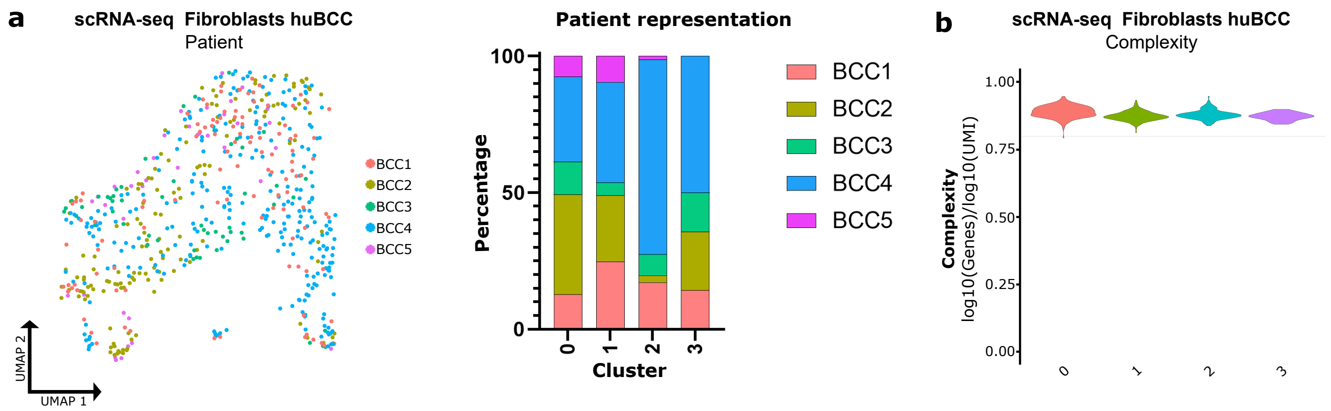
a
scRNA-seq Fibroblasts huBCC
Patient
BCC1
BCC2
BCC3
BCC4
BCC5
UMAP 2
UMAP 1
Patient representation
100
50
0
Percentage
0
1
2
3
Cluster
BCC1
BCC2
BCC3
BCC4
BCC5
b
scRNA-seq Fibroblasts huBCC
Complexity
Complexity
log10(Genes)/log10(UMI)
1.00
0.75
0.50
0.25
0.00
0
1
2
3

Supplementary Fig. 10 Quality control of the scRNA-seq fibroblast subpopulations.

a) UMAP plot of the fibroblast subpopulations colored according to huBCC samples (left panel) and bar graph of the scRNA-seq fibroblast subpopulations illustrating the contribution by sample to each cluster (right panel). **b)** Complexity of gene expression in each cluster from the scRNA-seq fibroblast subpopulations, represented as violin plots. Dotted line indicates the expected inferior threshold for satisfactory complexity (0.8).

scRNA-seq, single-cell RNA sequencing; huBCC, human basal cell carcinoma; BCC, basal cell carcinoma; UMAP, uniform manifold approximation and projection; UMI, unique molecular identifier.

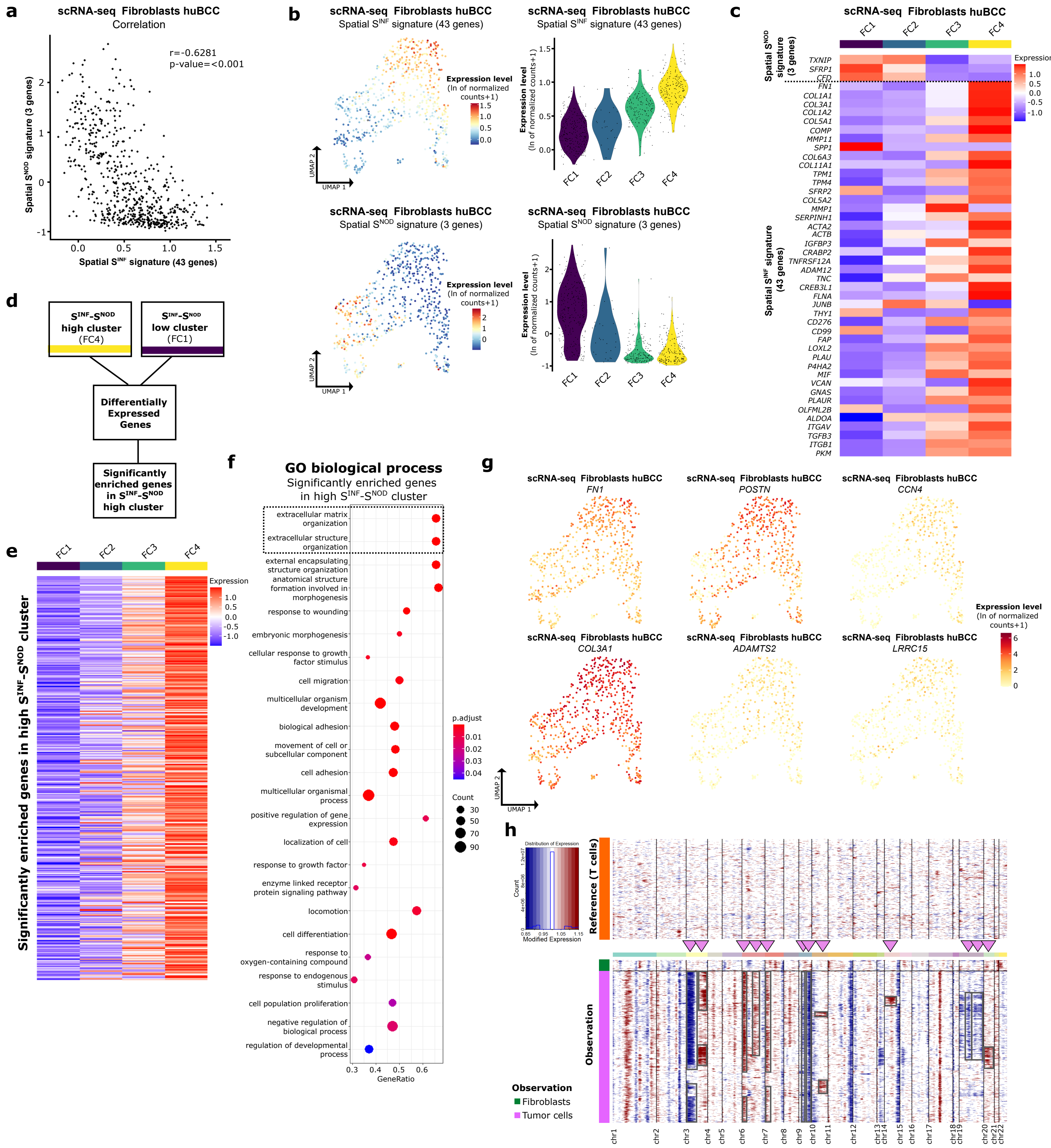
a
scRNA-seq Fibroblasts huBCC
Correlation
r=-0.6281
p-value=<0.001
Spatial S^NOD signature (3 genes)
Spatial S^INF signature (43 genes)
b
scRNA-seq Fibroblasts huBCC
Spatial S^INF signature (43 genes)
Expression level
(ln of normalized counts+1)
Spatial S^NOD signature (3 genes)
UMAP 1
UMAP 2
FC1
FC2
FC3
FC4
c
scRNA-seq Fibroblasts huBCC
Expression
Spatial S^NOD signature (3 genes)
TXNIP
SFRP1
CFD
Spatial S^INF signature (43 genes)
FN1
COL1A1
COL3A1
COL1A2
COL5A1
COMP
MMP11
SPP1
COL6A3
COL11A1
TPM1
TPM4
SFRP2
COL5A2
MMP1
SERPINH1
ACTA2
ACTB
IGFBP3
CRABP2
TNFRSF12A
ADAM12
TNC
CREB3L1
FLNA
JUNB
THY1
CD276
CD99
FAP
LOXL2
PLAU
P4HA2
MIF
VCAN
GNAS
PLAUR
OLFML2B
ALDOA
ITGAV
TGFB3
ITGB1
PKM
d
S^INF-S^NOD high cluster (FC4)
S^INF-S^NOD low cluster (FC1)
Differentially Expressed Genes
Significantly enriched genes in S^INF-S^NOD high cluster
e
Significantly enriched genes in high S^INF-S^NOD cluster
Expression
f
GO biological process
Significantly enriched genes in high S^INF-S^NOD cluster
extracellular matrix organization
extracellular structure organization
external encapsulating structure organization
anatomical structure formation involved in morphogenesis
response to wounding
embryonic morphogenesis
cellular response to growth factor stimulus
cell migration
multicellular organism development
biological adhesion
movement of cell or subcellular component
cell adhesion
multicellular organismal process
positive regulation of gene expression
localization of cell
response to growth factor
enzyme linked receptor protein signaling pathway
locomotion
cell differentiation
response to oxygen-containing compound
response to endogenous stimulus
cell population proliferation
negative regulation of biological process
regulation of developmental process
GeneRatio
p.adjust
Count
g
scRNA-seq Fibroblasts huBCC
FN1
POSTN
CCN4
COL3A1
ADAMTS2
LRRC15
Expression level
(ln of normalized counts+1)
h
Distribution of Expression
Modified Expression
Reference (T cells)
Observation
Fibroblasts
Tumor cells

Supplementary Fig. 11 Characterization of the fibroblast subpopulations within the invasive niche.

a) Correlation analysis between the spatial S^{INF} and spatial S^{NOD} signature enrichment scores in individual fibroblasts from the scRNA-seq fibroblast subpopulations. R coefficient and p-value were calculated by Spearman correlation test and a two-sided statistical significance test respectively. **b)** Average expression level of the spatial S^{INF} signature (upper panels) and spatial S^{NOD} signature (lower panels) in fibroblast subpopulations on the scRNA-seq dataset, represented as a color scale overlaid on the UMAP plot (left panels) and as violin plots per ranked cluster (right panels). **c)** Heatmap of the average expression level of genes present in the S^{NOD} and S^{INF} signatures in fibroblast subpopulations. **d)** Graphical representation of the establishment of the significantly enriched genes in clusters with high S^{INF}-S^{NOD} enrichment score in fibroblast subpopulations of the scRNA-seq dataset. **e)** Heatmap of the average expression level of significantly enriched genes in fibroblast subpopulations. **f)** Dot plot showing the GO biological processes enriched in the significantly enriched genes. **g)** Expression level of known infiltrative BCC-specific marker genes in fibroblast subpopulations of the scRNA-seq dataset, represented as a color scale overlaid on the UMAP plot. **h)** Representative CNV heatmap from inferCNV analysis of tumor cell and fibroblast subpopulations compared to T cells as reference (BCC1). Arrowheads and rectangles highlight gene expression changes that were not shared by the *KRT14*^{pos} epithelial cells from the five BCC samples and thus confidently representing genomic structural changes rather than cell lineage- or cluster-dependent transcriptional programs.

scRNA-seq, single-cell RNA sequencing; huBCC, human basal cell carcinoma; S^{NOD}, nodular stroma; S^{INF}, infiltrative stroma; UMAP, uniform manifold approximation and

projection; FC, fibroblast cluster; GO, gene ontology; p.adjust, adjusted p-value; chr, chromosome.

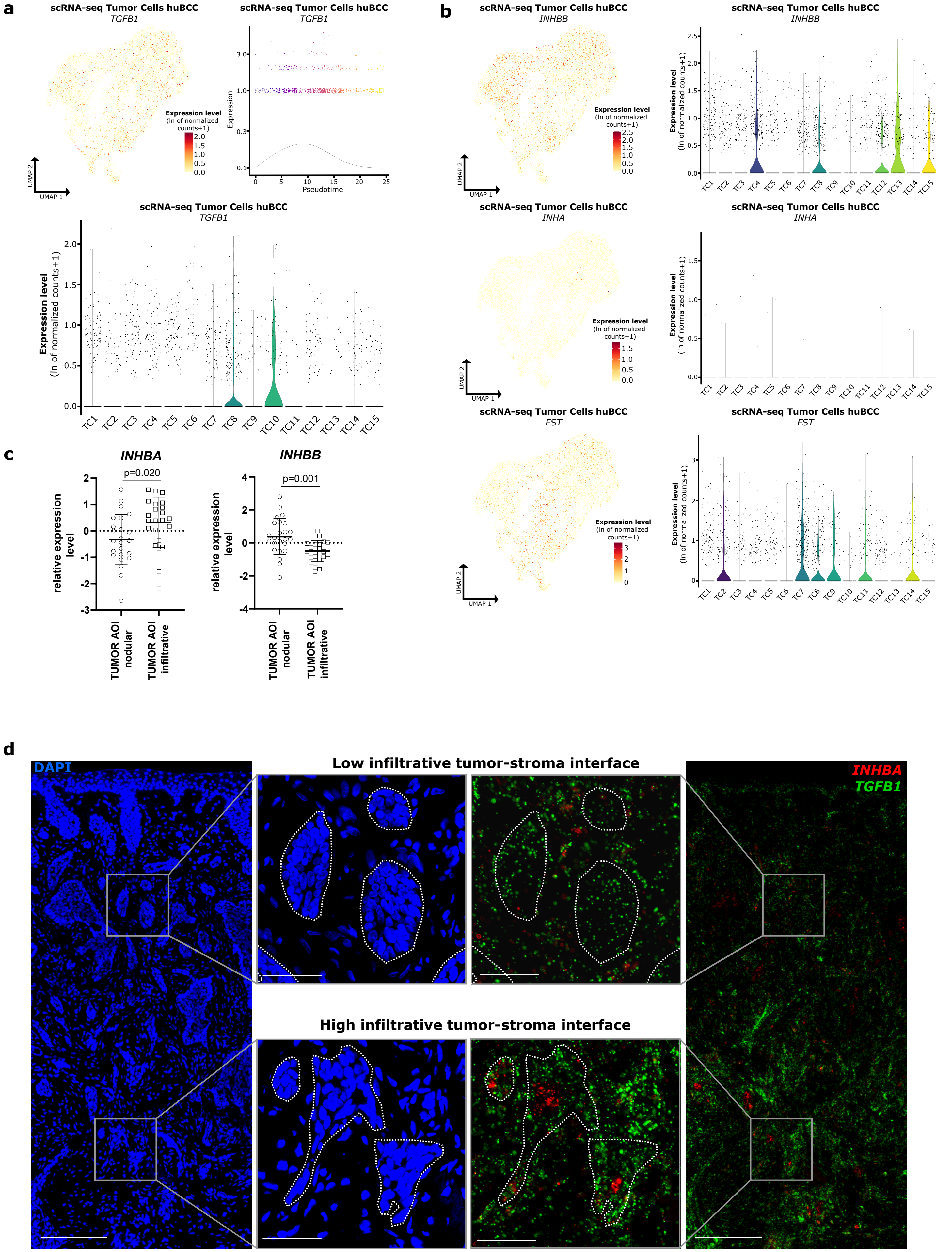

a
scRNA-seq Tumor Cells huBCC
TGFB1
Expression level
(ln of normalized counts+1)
2.0
1.5
1.0
0.5
0.0
UMAP 1
UMAP 2
scRNA-seq Tumor Cells huBCC
TGFB1
Expression
3.0
1.0
0.3
0.1
0
5
10
15
20
25
Pseudotime
scRNA-seq Tumor Cells huBCC
TGFB1
Expression level
(ln of normalized counts+1)
TC1
TC2
TC3
TC4
TC5
TC6
TC7
TC8
TC9
TC10
TC11
TC12
TC13
TC14
TC15
b
scRNA-seq Tumor Cells huBCC
INHBB
Expression level
(ln of normalized counts+1)
2.5
2.0
1.5
1.0
0.5
0.0
scRNA-seq Tumor Cells huBCC
INHBB
scRNA-seq Tumor Cells huBCC
INHA
scRNA-seq Tumor Cells huBCC
INHA
scRNA-seq Tumor Cells huBCC
FST
scRNA-seq Tumor Cells huBCC
FST
c
INHBA
p=0.020
relative expression level
TUMOR AOI nodular
TUMOR AOI infiltrative
INHBB
p=0.001
d
DAPI
Low infiltrative tumor-stroma interface
High infiltrative tumor-stroma interface
INHBA
TGFB1

Supplementary Fig. 12 *INHBA* and *TGFB1* show distinct expression patterns in infiltrative BCCs.

a) Expression level of *TGFB1* in tumor cell subpopulations of the scRNA-seq dataset, represented as a color scale overlaid on the UMAP plot (upper left panel) and as violin plots per ranked cluster (lower panel). Expression level per cell of *TGFB1* along the pseudotemporal trajectory of tumor cell subpopulations(upper right panel). **b)** Expression level of *INHBB, INHA* and *FST* in tumor cell subpopulations of the scRNA-seq dataset, represented as a color scale overlaid on the UMAP plot (left panels) and as violin plots per ranked cluster (right panels). **c)** Relative expression levels of *INHBA* and *INHBB* in nodular and infiltrative AOIs obtained from DSP. Horizontal bars represent the mean +/- SD. P-values calculated using unpaired two-sided Student's t-test. **d)** Representative scan images of an infiltrative BCC section stained with DAPI (blue), *INHBA* (red) and *TGFB1* (green) probes of RNA FISH. Upper and lower zoom areas show tumor-stroma interfaces with distinct Low and High infiltrative morphologies respectively. Dotted lines highlight the tumor-stroma interface. Scale bars indicate 200 μm (main panels) and 50 μm (zoom panels).

scRNA-seq, single-cell RNA sequencing; huBCC, human basal cell carcinoma; UMAP, uniform manifold approximation and projection; TC, tumor cluster; AOI, area of interest.

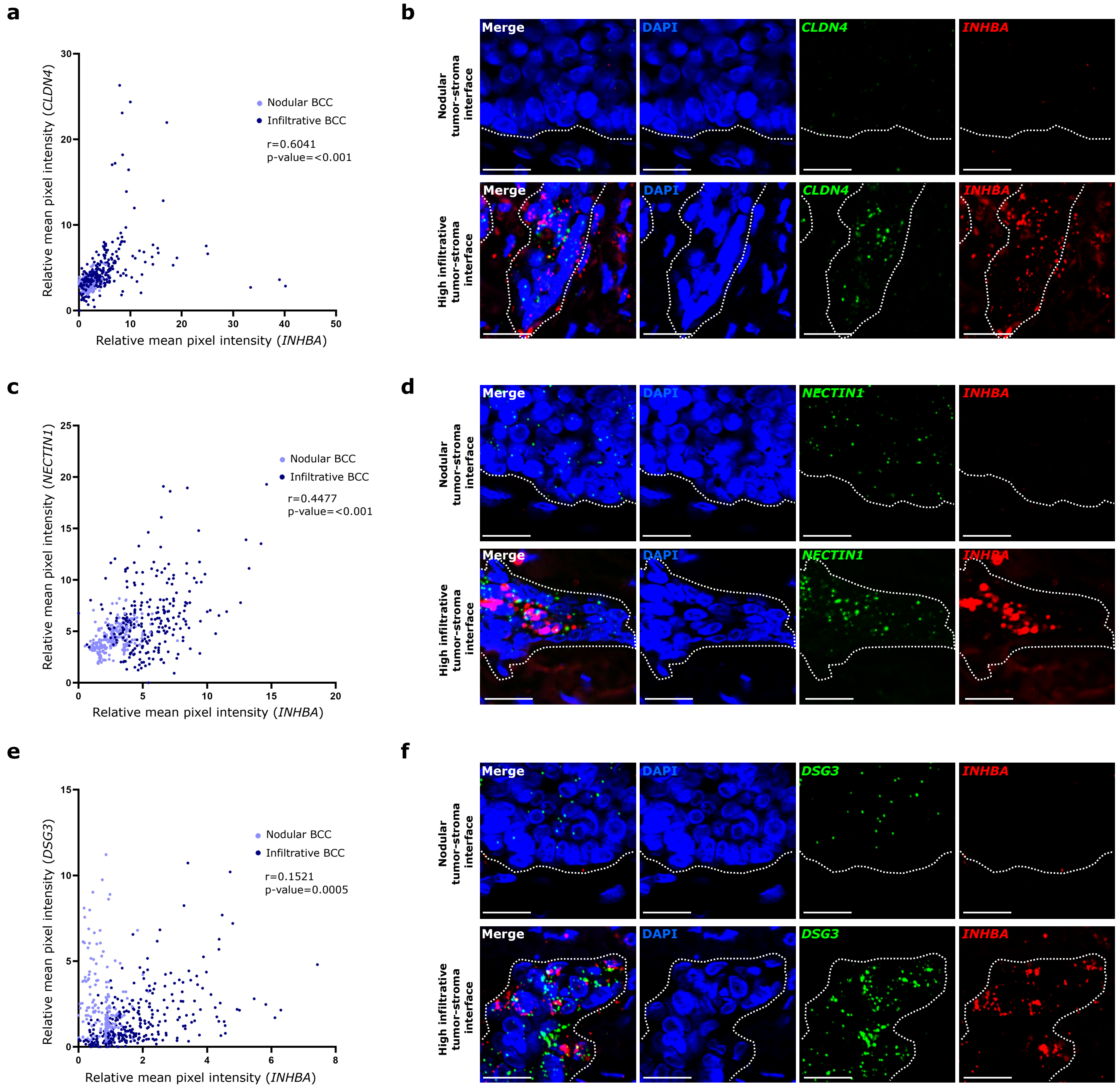

a
b
c
d
e
f
Relative mean pixel intensity (CLDN4)
Relative mean pixel intensity (INHBA)
Nodular BCC
Infiltrative BCC
r=0.6041
p-value=<0.001
Relative mean pixel intensity (NECTIN1)
r=0.4477
p-value=<0.001
Relative mean pixel intensity (DSG3)
r=0.1521
p-value=0.0005
Merge
DAPI
CLDN4
INHBA
NECTIN1
DSG3
Nodular tumor-stroma interface
High infiltrative tumor-stroma interface

Supplementary Fig. 13 *INHBA* expression correlates with marker genes of collective migration in highly infiltrative tumor cells.

a) Correlation analysis between tumor *CLDN4* and *INHBA* relative intensities at the tumor-stroma interface in nodular (N=4) and infiltrative (N=5) BCCs (n≥45 regions/sample). **b)** Representative scan images of Nodular and High infiltrative tumor-stroma interfaces stained with DAPI (blue), *CLDN4* (green) and *INHBA* (red) probes for RNA FISH. **c)** Correlation analysis between tumor *NECTIN1* and *INHBA* relative intensities at the tumor-stroma interface in nodular (N=4) and infiltrative (N=4) BCCs (n≥45 regions/sample). **d)** Representative scan images of Nodular and High infiltrative tumor-stroma interfaces stained with DAPI (blue), *NECTIN1* (green) and *INHBA* (red) probes for RNA FISH. **e)** Correlation analysis between tumor *DSG3* and *INHBA* relative intensities at the tumor-stroma interface in nodular (N=4) and infiltrative (N=5) BCCs (n≥45 regions/sample). **f)** Representative scan images of Nodular and High infiltrative tumor-stroma interfaces stained with DAPI (blue), *DSG3* (green) and *INHBA* (red) probes for RNA FISH.

R coefficients and p-values in a), c) and e) were calculated by Spearman correlation test and a two-sided statistical significance test respectively. In panels b), d) and f), dotted lines highlight the tumor-stroma interface and scale bars indicate 20 μm.

Source data for a), c) and e) are provided as a Source Data file.

BCC, basal cell carcinoma.

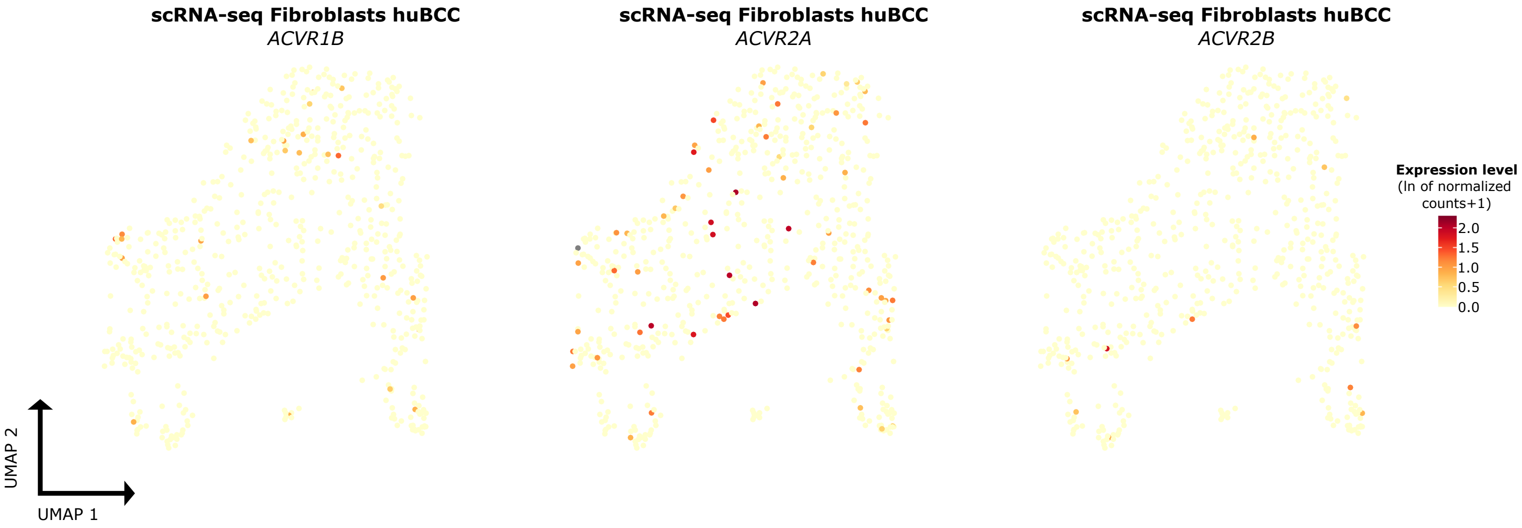
scRNA-seq Fibroblasts huBCC
ACVR1B
scRNA-seq Fibroblasts huBCC
ACVR2A
scRNA-seq Fibroblasts huBCC
ACVR2B
Expression level
(ln of normalized counts+1)
2.0
1.5
1.0
0.5
0.0
UMAP 2
UMAP 1

Supplementary Fig. 14 Activin A receptors expression in CAFs.

Expression level of Activin A receptor genes (*ACVR1B, ACVR2A* and *ACVR2B*) in fibroblast subpopulations of the scRNA-seq dataset, represented as a color scale overlaid on the UMAP plot.

scRNA-seq, single-cell RNA sequencing; huBCC, human basal cell carcinoma; UMAP, uniform manifold approximation and projection; CAFs, cancer-associated fibroblasts.

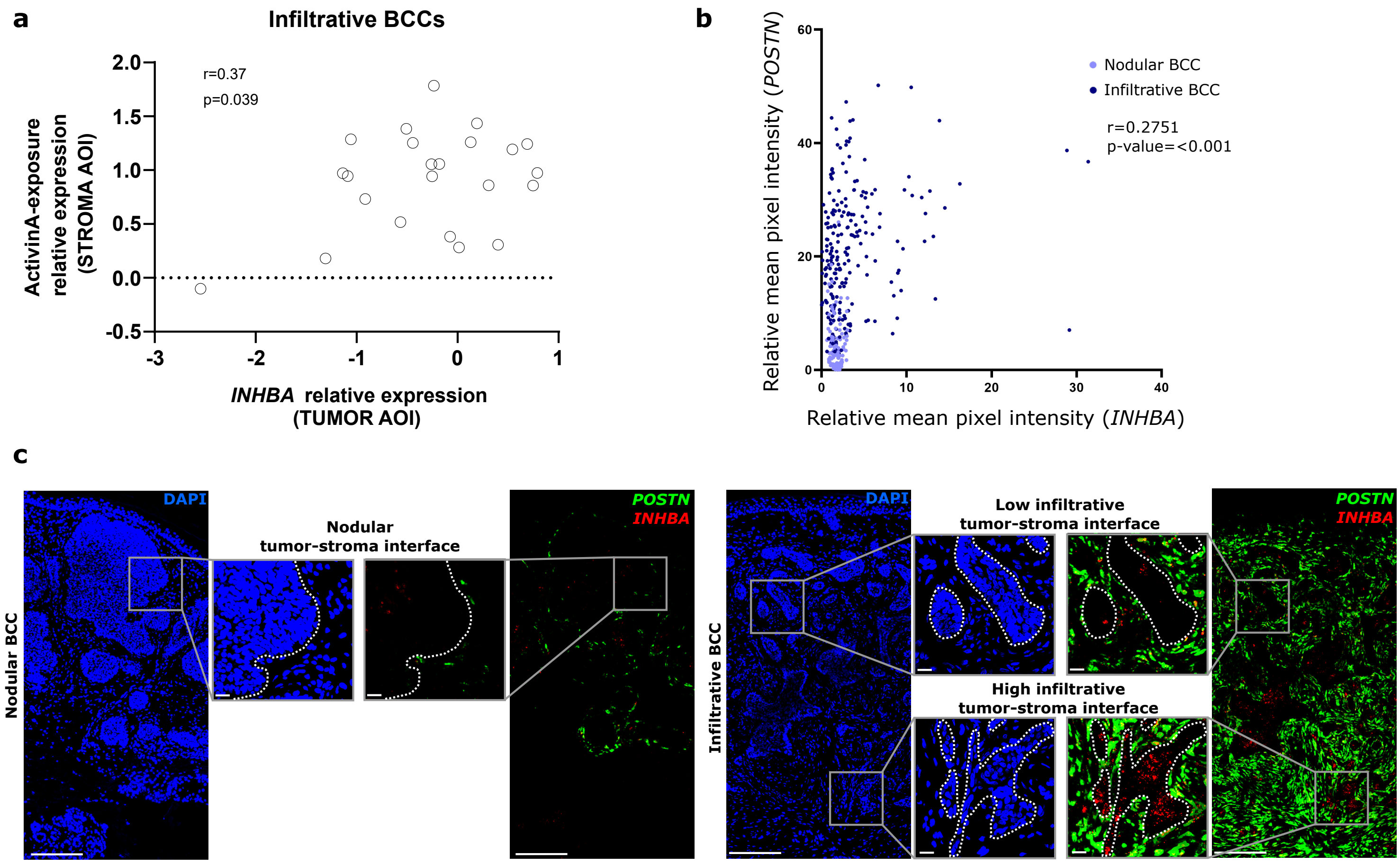
a
Infiltrative BCCs
r=0.37
p=0.039
ActivinA-exposure relative expression (STROMA AOI)
INHBA relative expression (TUMOR AOI)
b
Nodular BCC
Infiltrative BCC
r=0.2751
p-value=<0.001
Relative mean pixel intensity (POSTN)
Relative mean pixel intensity (INHBA)
c
DAPI
Nodular tumor-stroma interface
POSTN
INHBA
Nodular BCC
DAPI
Low infiltrative tumor-stroma interface
High infiltrative tumor-stroma interface
POSTN
INHBA
Infiltrative BCC

Supplementary Fig. 15 *INHBA* expression in tumor correlates with ECM genes expression in surrounding CAFs.

a) Correlation analysis between the expression of *INHBA* in tumor AOI and Activin A-exposure signature (*FN1, COL1A1, ACTA2, IL6, SPP1*) in adjacent stroma AOI of infiltrative BCCs, based on DSP. **b)** Correlation analysis between tumor *INHBA* and adjacent stromal *POSTN* relative intensities in nodular (N=4) and infiltrative (N=8) BCCs (n≥15 regions/sample). **c)** Representative scan images of nodular and infiltrative BCC sections stained with DAPI (blue), *INHBA* (red) and *POSTN* (green) probes of RNA FISH. Upper, intermediate and lower zoom areas show tumor-stroma interfaces with distinct Nodular, Low and High infiltrative morphologies respectively. Dotted lines highlight the tumor-stroma interface. Scale bars indicate 200 µm (main panels) and 20 µm (zoom panels).

R coefficients and p-values in a) and b) were calculated by Spearman correlation test and a two-sided statistical significance test respectively.

Source data for b) are provided as a Source Data file.

AOI, area of interest; BCC, basal cell carcinoma; ECM, extracellular matrix; CAF, cancer-associated fibroblast.
