# Supplementary material for: Integrated multi-omics reveals cellular and molecular interactions governing the invasive niche of basal cell carcinoma
Source: Nat Commun. 2022 Aug 20;13:4897. doi: 10.1038/s41467-022-32670-w (PMC9391376; doi:10.1038/s41467-022-32670-w)
Supplement: Supplementary file 3 — Description of Additional Supplementary Files [file 41467_2022_32670_MOESM3_ESM.pdf]

Description of Additional Supplementary Files

File Name: Supplementary Data 1
Description: Differentially expressed genes in infiltrative versus nodular Tumor AOIs.

File Name: Supplementary Data 2
Description: Differentially expressed genes in infiltrative versus nodular Stroma AOIs.

File Name: Supplementary Data 3
Description: Compartment-specific signatures for nodular and infiltrative tumorstroma interfaces.

File Name: Supplementary Data 4
Description: Differentially expressed genes between clusters with high TINFTNOD (TC12, TC13, TC14, TC15) and clusters with low TINF-TNOD (TC1, TC2, TC3, TC4) enrichment scores.

File Name: Supplementary Data 5
Description: Differentially expressed genes between cluster with high SINFSNOD (FC4) and cluster with low SINF-SNOD (FC1) enrichment scores.

File Name: Supplementary Data 6
Description: Moran's I pseudotime autocorrelation indexes.

File Name: Supplementary Data 7
Description: Signatures applied to the scRNAseq dataset.
