# Supplementary material for: Integrated multi-omics reveals cellular and molecular interactions governing the invasive niche of basal cell carcinoma
Source: Nat Commun. 2022 Aug 20;13:4897. doi: 10.1038/s41467-022-32670-w (PMC9391376; doi:10.1038/s41467-022-32670-w)
Supplement: Supplementary file 11 — Reporting Summary [file 41467_2022_32670_MOESM11_ESM.pdf]

nature portfolio

Corresponding author(s): François Kuonen
Last updated by author(s): Aug 6, 2022

Reporting Summary

Nature Portfolio wishes to improve the reproducibility of the work that we publish. This form provides structure for consistency and transparency in reporting. For further information on Nature Portfolio policies, see our Editorial Policies and the Editorial Policy Checklist.

Statistics

For all statistical analyses, confirm that the following items are present in the figure legend, table legend, main text, or Methods section.

n/a	Confirmed	
☐	☒	The exact sample size (*n*) for each experimental group/condition, given as a discrete number and unit of measurement
☐	☒	A statement on whether measurements were taken from distinct samples or whether the same sample was measured repeatedly
☐	☒	The statistical test(s) used AND whether they are one- or two-sided<br>*Only common tests should be described solely by name; describe more complex techniques in the Methods section.*
☒	☐	A description of all covariates tested
☒	☐	A description of any assumptions or corrections, such as tests of normality and adjustment for multiple comparisons
☐	☒	A full description of the statistical parameters including central tendency (e.g. means) or other basic estimates (e.g. regression coefficient) AND variation (e.g. standard deviation) or associated estimates of uncertainty (e.g. confidence intervals)
☐	☒	For null hypothesis testing, the test statistic (e.g. *F*, *t*, *r*) with confidence intervals, effect sizes, degrees of freedom and *P* value noted<br>*Give P values as exact values whenever suitable.*
☒	☐	For Bayesian analysis, information on the choice of priors and Markov chain Monte Carlo settings
☒	☐	For hierarchical and complex designs, identification of the appropriate level for tests and full reporting of outcomes
☒	☐	Estimates of effect sizes (e.g. Cohen's *d*, Pearson's *r*), indicating how they were calculated

Our web collection on statistics for biologists contains articles on many of the points above.

Software and code

Policy information about availability of computer code

Data collection	single-cell RNA sequencing: Paired-end sequencing was performed on Illumina HiSeq 4000 device (Hiseq Control Software, v.3.4.0) for BCC1 and BCC2 and on NovaSeq 10x device (Novaseq Control Software v.1.7.5) for BCC3, BCC4 and BCC5.<br><br>Digital spatial profiling: Selection of the regions of interest (ROIs) was performed with the GeoMx DSP analysis suite version 2.2.0.111 (Nanostring). Paired-end sequencing was performed on an Illumina HiSeq4000 instrument (Hiseq Control Software, v.3.4.0)<br><br>For the rest: RNA FISH, the slides were acquired with Panoramic 250 slide scanner. For immunofluorescence and FISH co-detection, the slides were acquired with Zeiss LSM 700 confocal microscope.
Data analysis	single-cell RNA sequencing: Sequencing data were demultiplexed using the bcl2fastq2 Conversion Software (v. 2.20, Illumina). Fastq files of raw sequencing data were processed using the count function of the Cell Ranger pipeline (v. 5.0.1, 10x Genomics). The count function allowed us to demultiplex sequencing reads to individual cells, to align the reads to the human GRCh38 genome reference (v. GRCh38-2020-A), and to generate filtered matrices of gene counts by cell barcodes. The expression matrix was analyzed in R (v. 4.1.0) language with these packages: Seurat (v. 4.0.4), lisi (v.1.0), clusterProfiler (v.4.0.5), org.Hs.eg.db (v.3.13.0), stats (v.4.1.0),infercnv (v.1.8.1), monocle3 (v.1.0.0).<br><br>Digital spatial profiling: Spatial data were processed by GeoMx DSP analysis suite (v.2.2.0.111). HiSeq-derived FASTQ files for each sample were compiled for each compartment using the bcl2fastq Conversion Software (v. 2.20, Illumina) and then demultiplexed and converted to digital count conversion (DCC) files using the GeoMx DnD pipeline (v.1) of Nanostring according to manufacturer's pipeline. DCC files were imported back into the DSP instrument for QC and data analysis using GeoMx DSP analysis suite version 2.2.0.111 (Nanostring).<br><br>Images were processed with ZEN 2.3 lite and (Fiji Is Just) Image J.<br><br>The software used for statistical analyses are GraphPad Prism (version 8.3.0) and GeoMx DSP analysis suite (version 2.2.0.111, Nanostring)

For manuscripts utilizing custom algorithms or software that are central to the research but not yet described in published literature, software must be made available to editors and reviewers. We strongly encourage code deposition in a community repository (e.g. GitHub). See the Nature Portfolio guidelines for submitting code & software for further information.

Data

Policy information about availability of data

All manuscripts must include a data availability statement. This statement should provide the following information, where applicable:

- Accession codes, unique identifiers, or web links for publicly available datasets
- A description of any restrictions on data availability
- For clinical datasets or third party data, please ensure that the statement adheres to our policy

The scRNA-seq data generated in this study have been deposited in the Gene Expression Omnibus database under accession code GSE181907 (https://www.ncbi.nlm.nih.gov/geo/query/acc.cgi?acc=GSE181907). The DSP data generated in this study have been deposited in the Gene Expression Omnibus database under accession code GSE210648 (https://www.ncbi.nlm.nih.gov/geo/query/acc.cgi?acc=GSE210648). The following databases and datasets were used in this study: GRCh38 human reference genome reference (refdata-gex-GRCh38-2020-A) (https://support.10xgenomics.com/single-cell-gene-expression/software/downloads/latest), GeoMx® Cancer Transcriptome Atlas (https://www.nanostring.com/products/geomx-digital-spatial-profiler/geomx-rna-assays/geomx-cancer-transcriptome-atlas/).
The remaining data are available within the Supplementary Information or Source Data file.
Source data are provided with this paper.
There is no restriction in data availability.

Field-specific reporting

Please select the one below that is the best fit for your research. If you are not sure, read the appropriate sections before making your selection.

☒ Life sciences ☐ Behavioural & social sciences ☐ Ecological, evolutionary & environmental sciences

For a reference copy of the document with all sections, see nature.com/documents/nr-reporting-summary-flat.pdf

Life sciences study design

All studies must disclose on these points even when the disclosure is negative.

Sample size	For scRNA-seq, we obtained 5 samples of infiltrative BCC. The sample sizes were sufficient as we obtained thousands of cells in each sample, with satisfatory integration.<br>For digital spatial profiling, we obtained n≥23 biological replicates per condition (distributed over 6 nodular and 6 infiltrative BCCs). The sample sizes were sufficient as we obtained specific spatial transcriptomic signatures.<br>For RNA FISH/immunostaining, we included n≥4 samples per disease condition. Samples sizes were chosen according to the standards of the field (generally ≥3 biological replicates for each condition).
Data exclusions	We did not exclude any sample.
Replication	The samples in the same disease condition can be considered as biological replicates.<br>For scRNA-seq, we have 5 biological replicates. The replications were successful as the biological replicates showed similar gene expression profiles and satisfactory integration.<br>For DSP, we have n≥23 biological replicates per condition (distributed over 6 nodular and 6 infiltrative BCCs). The sample sizes were sufficient as we obtained specific spatial transcriptomic signatures.<br>For RNA FISH/immunostaining, the biological replicates were clearly stated for each experiment in the figure legend. Where statistical comparisons were made, experiments were performed with n≥4 independent biological replicates to ensure reproducibility.
Randomization	For RNA FISH/immunostaining, for comparison between the disease groups, fields of view were chosen randomly at the tumor-stroma interface after a scan through the entire tissue.
Blinding	For RNA FISH/immunostaining: blinding for comparison between experimental conditions was not possible as disease categroy was evident from image data.<br>For scRNA-seq and DSP, our goal was not to predict wether a sample was part of the disease or control group, but rather to study differences between different disease groups and therefore blinding could not be applied.

Reporting for specific materials, systems and methods

We require information from authors about some types of materials, experimental systems and methods used in many studies. Here, indicate whether each material, system or method listed is relevant to your study. If you are not sure if a list item applies to your research, read the appropriate section before selecting a response.

Materials & experimental systems

n/a	Involved in the study
☐	☒ Antibodies
☒	☐ Eukaryotic cell lines
☒	☐ Palaeontology and archaeology
☒	☐ Animals and other organisms
☐	☒ Human research participants
☒	☐ Clinical data
☒	☐ Dual use research of concern

Methods

n/a	Involved in the study
☒	☐ ChIP-seq
☒	☐ Flow cytometry
☒	☐ MRI-based neuroimaging

Antibodies

Antibodies used

panCK-532 (clone AE1+AE3) (Novus, NBP2-33200)(dilution 1:20), Monoclonal antibody to human cytokeratin (pan)(clone Lu-5) (BMA BIOMEDICALS, T-1302) (dilution 1:250), F(ab')2-Goat anti-Mouse IgG (H+L) Cross-Adsorbed Secondary Antibody, Alexa Fluor 488 (Life Technologies, A11017) (dilution 1:500)

Validation

panCK antibody (Nanostring Technologies): https://www.nanostring.com/products/geomx-digital-spatial-profiler/geomx-dsp-overview. Merritt CR et al, Multiplex digital spatial profiling of proteins and RNA in fixed tissue. Nature Biotechnology volume 38, pages586–599 (2020). Validated for GeoMXTM Digital Spatial Profiling.
Monoclonal antibody to human cytokeratin (pan) (clone Lu-5)(BMA BIOMEDICALS): http://www.bma.ch/files/product/t-1302-lot-20.pdf
F(ab')2-Goat anti-Mouse IgG (H+L) Cross-Adsorbed Secondary Antibody, Alexa Fluor 488 (Life Technologies): https://www.thermofisher.com/order/genome-database/dataSheetPdf?producttype=antibody&productsubtype=antibody_secondary&productId=A-11017&version=194

Human research participants

Policy information about studies involving human research participants

Population characteristics

single-cell RNA sequencing samples:
- BCC1: infiltrative BCC, left ear, no previous treatment
- BCC2: infiltrative BCC, forehead, no previous treatment
- BCC3: infiltrative BCC, left cheek, no previous treatment
- BCC4: infiltrative BCC, nose, no previous treatment
- BCC5: infiltrative BCC, nose, no previous treatment

Digital spatial profiling samples:
- DSP1: nodular BCC, right leg, no previous treatment
- DSP2: nodular BCC, scalp, no previous treatment
- DSP3: nodular BCC, left leg, no previous treatment
- DSP4: nodular BCC, upper lip, no previous treatment
- DSP5: nodular BCC, forehead, no previous treatment
- DSP6: nodular BCC, right cheek, no previous treatment
- DSP7: infiltrative BCC, left eyebrow, no previous treatment
- DSP8: infiltrative BCC, right elbow, no previous treatment
- DSP9: infiltrative BCC, left ear, no previous treatment
- DSP10: infiltrative BCC, left ear, no previous treatment
- DSP11: infiltrative BCC, left cheek, no previous treatment
- DSP12: infiltrative BCC, nost, no previous treatment

Recruitment

Residual tissue left after standard histopatholgical analysis of surgical excisions were collected. No perceived selection bias. Sample availability was dependent on patients willingness to participate in the study, and obtention of a written informed consent.

Ethics oversight

Studies were approved by the institutional review board of Lausanne University Hospital CHUV, and the local ethics committee, in accordance with the Helsinki Declaration (CER-VD 2020-02204). Written informed consent was obtained from each patient.

Note that full information on the approval of the study protocol must also be provided in the manuscript.
